# UPLC-MS-ESI-QTOF Analysis and Antifungal Activity of the *Spondias tuberosa* Arruda Leaf and Root Hydroalcoholic Extracts

**DOI:** 10.3390/antibiotics8040240

**Published:** 2019-11-28

**Authors:** Antonia Thassya Lucas dos Santos, Joara Nályda Pereira Carneiro, Rafael Pereira da Cruz, Débora Lima Sales, Jacqueline Cosmo Andrade, Waltécio de Oliveira Almeida, José Galberto Martins da Costa, Paulo Riceli Vasconcelos Ribeiro, Edy Sousa de Brito, Francisco Lucas Alves Batista, Francisco Ernani Alves Magalhães, Marcello Iriti, Maria Flaviana Bezerra Morais-Braga, Henrique Douglas Melo Coutinho

**Affiliations:** 1 Department of Biological Chemistry, Regional University of Cariri-URCA, Col Antonio Luis, 1161, 63105-000 Pimenta, Crato-CE, Brazil; thassyalucas@hotmail.com (A.T.L.d.S.); nalyda_05@hotmail.com (J.N.P.C.); rafaelcruz284@gmail.com (R.P.d.C.); debora.lima.sales@gmail.com (D.L.S.); waltecio@gmail.com (W.d.O.A.); galberto.martins@gmail.com (J.G.M.d.C.); flucasbatista@outlook.com (F.L.A.B.); flavianamoraisb@yahoo.com.br (M.F.B.M.-B.); hdmcoutinho@gmail.com (H.D.M.C.); 2 Brejo Santo Campus, Federal University of Cariri-UFCA, R. Olegario Emidio de Araujo, s/n, 63260-000 Centro, Brejo Santo-CE, Brazil; jacqueline.andrade@ufca.edu.br; 3 Embrapa Tropical Agroindustry, R. Pernambuco, 2270, 60511-110 Pici, Fortaleza-CE, Brazil; pauloriceli85@gmail.com (P.R.V.R.); edy.brito@embrapa.br (E.S.d.B.); 4 State University of Ceara-Tauá Campus, BR-020-Bezerra de Sousa, 63660-000 Tauá-CE, Brazil; fernanimagalhaes@yahoo.com.br; 5 Department of Agricultural and Environmental Sciences, Milan State University, via G. Celoria 2, 20133 Milan, Italy

**Keywords:** chromatography, fluconazole, *Candida* spp., morphological transition

## Abstract

The aim of this study was to identify and evaluate the chemical compositions and effects of the *S. tuberosa* leaf and root hydroalcoholic extracts (HELST and HERST) against different strains of *Candida*. Chemical analysis was performed by Ultra-Performance Liquid Chromatography Coupled to Quadrupole/Time of Flight System (UPLC-MS-ESI-QTOF). The Inhibitory Concentration of 50% of the growth (IC_50)_ as well as the intrinsic and combined action of the extracts with the antifungal fluconazole (FCZ) were determined by the microdilution method while the minimum fungicidal concentrations (MFCs) and the effect on fungal morphological transitions were analyzed by subculture and in humid chambers, respectively. From the preliminary phytochemical analysis, the phenols and flavonoids were the most abundant. The intrinsic IC_50_ values for HELST ranged from 5716.3 to 7805.8 µg/mL and from 6175.4 to 51070.9 µg/mL for the HERST, whereas the combination of the extracts with fluconazole presented IC_50_ values from 2.65 to 278.41 µg/mL. The MFC of the extracts, individually, for all the tested strains was ≥16384 µg/mL. When fluconazole was combined with each extract, the MFC against CA URM 5974 was reduced (HELST: 2048 and HERST: 4096 µg/mL). Synergism was observed against standard *C. albicans* (CA) and *C. tropicalis* (CT) strains and with the root extract against the CT isolate. The leaf extract inhibited the morphological transition of all strains while the root extract inhibited only CT strains.

## 1. Introduction

*Candida* species, especially *C. albicans*, are commonly found on human mucosal surfaces but are becoming an important progressive invasive pathogen due to their increased prevalence in immunocompromised patients and the increased use of antibiotics [1].

Infections caused by the *Candida* genus are associated with a high morbidity and mortality rate, where these species are responsible for superficial and systemic candidiasis, with the latter being a serious problem for health systems and patients [2]. Several *Candida* species are polymorphic and able to transition between different morphological conditions involving yeast, hyphal, and pseudohyphal forms [3].

The inappropriate use of antifungal drugs contributes to the increase in microbial resistance, thus an understanding the mechanisms of antifungal drugs, the cellular molecular mechanisms involved in the antifungal resistance process, is sought [4]. Given the few commercially available antifungals and their varied side effects, a need to produce new and more effective antifungal agents with fewer adverse effects exists [5].

Studies addressing the use of plants have been growing in Brazil as well as worldwide, increasing the importance and knowledge of their chemical components [6]. In this sense, natural products and their chemical diversity are seen as options for potentially active therapeutic sources, which can be a means of discovering new drugs [7].

*Spondias tuberosa* Arruda (umbu) is a native plant from Brazil, belonging to the Anacardiaceae family, with great ecological and economic importance [8], being used in popular medicine for infections, stomach disorders, and inflammatory conditions [9]. Among its biological activities, its anti-inflammatory [10], anticancer [11], antibacterial [12], antioxidant [13], and antiviral activities stand out [14].

Using ethnobiology as a guiding strategy for bioactivity research, the objective of this study was to identify the chemical composition of the *S. tuberosa* leaf and root hydroalcoholic extracts and their antifungal effect, both the intrinsic and combined effect with conventional fluconazole, as well as their action on morphological transition, a *Candida* spp. virulence factor.

## 2. Results

The leaf and root extract yields were, respectively, 0.74% and 0.635%. Preliminary phytochemical analysis of the extracts revealed the presence of several metabolite classes, such as: Alkaloids, steroids, phenols, flavonoids, triterpenoids, and xanthones (Table 1). It is noteworthy that the presence of phenols and flavonoids prevailed in all extracts. Among the secondary metabolites investigated, coumarins, flavanonols, and tannins were not detected.

In UPLC-MS-ESI-QTOF, identification of the compounds was based on their molecular ion mass, retention time, fragmentation pattern, and data available in the literature, as shown in Table 2 and Table 3, according to the order of elution, molecular formula, error, and major fragments (MS2).

Table 4 shows the IC_50_ values of the extracts, fluconazole, and their combined action, where the concentration in which the natural product or the drug could reduce the microorganismal population by 50% can be seen. The HESTL and HESTR values were not clinically significant while fluconazole achieved a 50% reduction in the fungal population, whereas when associated with leaf extract, there was an increase in drug action. With root extract, the effect was similar to fluconazole for *C. albicans* strains, and for CT INCQS 40042, there was a decrease in the effect of the drug, while for CT URM there was an improvement in fluconazole action.

**Figure 1:**
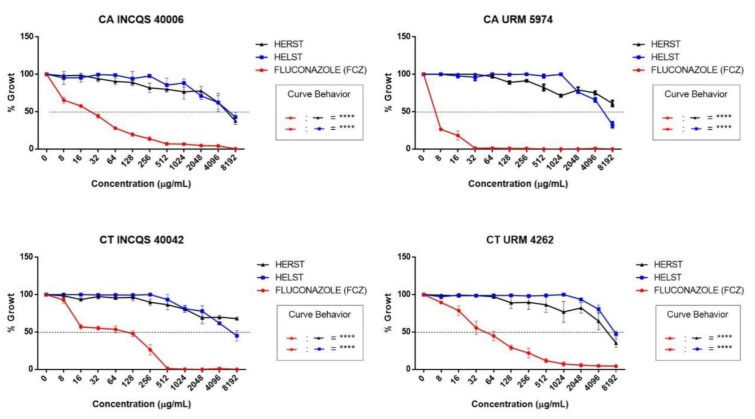
Cell viability curve demonstrating the inhibitory effect of fluconazole and hydroalcoholic extracts of *Spondias tuberosa* against *Candida* strains. CA: *Candida albicans*; CT: *Candida tropicalis*; INCQS: National Institute of Quality Control in Health; URM: University Recife Mycology; HELST: Hydroalcoholic Extract of Leaves of *Spondias tuberosa*; HERST: Hydroalcoholic Extract of the Roots of *Spondias tuberosa*; FCZ: Fluconazole. ****—Statistical significance with *p* < 0.0001.

The action of the extracts and fluconazole on the cell viability curve can be seen in Figure 1. The extracts presented no relevant action against the tested strains while fluconazole presented a more effective action against CA URM 5974 (32 µg/mL), and against other strains when its concentration was increased.

The MFC of the extracts against all tested strains was ≥16,384 µg/mL, as well as the fluconazole MFC against *C. tropicalis*, thus observing a fungistatic effect. The FCZ effect was termed fungicidal at a concentration of 8192 µg/mL (Table 5) against standard and isolated *C. albicans* strains. An MFC of 2048 and 4096 µg/mL, respectively, was observed against the CA isolate in the fluconazole combination with the HELST and HERST extracts while the MFC ranged from 8192 to ≥16,384 µg/mL against the other tested strains.

**Figure 2:**
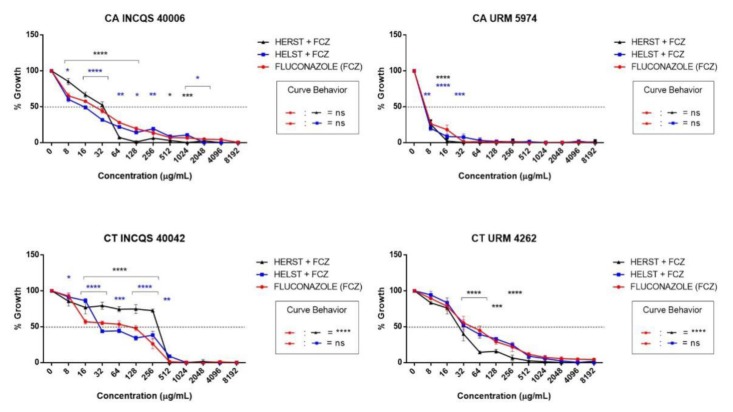
Evaluation of the modifying effect of fluconazole action by extracts. CA: *Candida albicans*; CT: *Candida tropicalis*; INCQS: National Institute of Quality Control in Health; URM: University Recife Mycology; HELST: Hydroalcoholic Extract of Leaves of *Spondias tuberosa*; HERST: Hydroalcoholic Extract of the Roots of *Spondias tuberosa*; FCZ: Fluconazole; NS: not significant; *—Statistical significance with *p* < 0.05; **—Statistical significance with *p* < 0.01; ***—Statistical significance with *p* < 0.001; ****—Statistical significance with *p* < 0.0001.

When combined with the antifungal agent fluconazole, the HELST potentiated the effect of the drug, where a synergistic effect against CA INCQS 40006 (8 to 256 µg/mL) and CT INCQS 40042 (8 to 512 µg/mL) was observed. On the other hand, the cell viability curve for CA URM 5974 and CT URM 4262 isolates was similar to that of fluconazole (Figure 2).

A synergism against CA INCQS 40006 (64 to 1024 µg/mL), CA URM 5974 (16 µg/mL), and CT URM 4262 (8 to 1024 µg/mL) can be observed when the HERST was combined with fluconazole. However, an antagonism was observed against the standard CT INCQS 40042 strain (16 to 256 µg/mL), where the combination with the extract diminished the effect of the drug.

**Figure 3:**
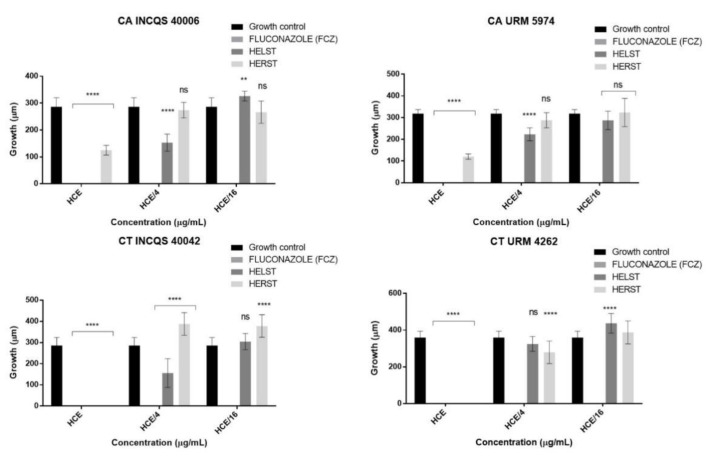
Effect of extracts and fluconazole on the morphological transition of *Candida* spp. CA: *Candida albicans*; CT: *Candida tropicalis*; INCQS: National Institute of Quality Control in Health; URM: University Recife Mycology; HELST: Hydroalcoholic Extract of Leaves of *Spondias tuberosa*; HERST: Hydroalcoholic Extract of the Roots of *Spondias tuberosa* FCZ: Fluconazole; HCE: Higher Concentration Evaluated; ns: not significant. NS: not significant; **—Statistical significance with *p* < 0.01; ****—Statistical significance with *p* < 0.0001.

In the antivirulence assay, the HELST was able to inhibit hyphal emission at the highest concentration (HCE) in all tested strains. At the HCE/4 concentration, the extract reduced morphological transition by 44.09% and 45.45%, respectively, against CA INCQS 40006 and CT INCQS 40042 when compared to the growth control. At lower concentrations, the HELST presented insignificant or similar results to the growth control. Fluconazole inhibited the tested strains at all concentrations (Figure 3).

The HERST caused filament inhibition only at the highest concentration for CT INCQS 40042 and CT URM 4262 while it reduced hyphal growth by 54.30% and 62.09% respectively, for CA INCQS 40006 and CA URM 5974, all relative to the growth control. At the lowest concentrations, the HERST did not obtain considerable results.

**Figure 4:**
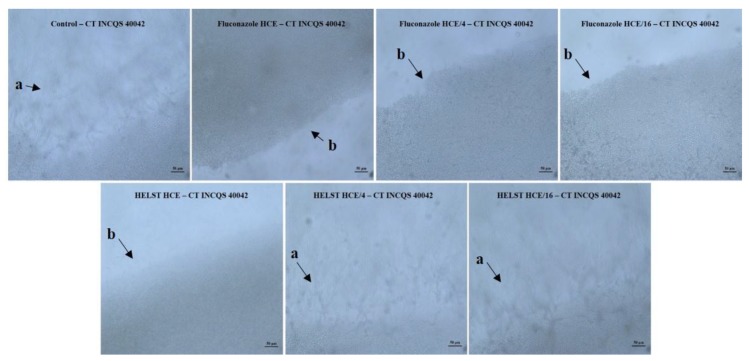
Demonstrative effect of HELST and fluconazole on the morphological transition of *Candida tropicalis*. CT: *Candida tropicalis*; INCQS: National Institute of Quality Control in Health; URM: University Recife Mycology; HELST: Hydroalcoholic Extract of Leaves of *Spondias tuberosa*; FCZ: Fluconazole; HCE: Higher Concentration Evaluate; a: presence of filamentous structures; b: absence of filamentous structures. A—hyphae development; B—hyphae inhibition.

Figure 4 shows the filamentous structures in the growth control and the progressive effect of fluconazole and HELST on *C. tropicalis* yeast morphology (CT INCQS 40042). The micromorphology recordings for fluconazole presented inhibition at all tested concentrations while the HELST caused inhibition at the HCE concentration (8192 µg/mL) and a decrease in hyphae and pseudohyphae growth at the HCE/4 concentration (2048 µg/mL). The HCE/16 concentration (512 µg/mL) presented similar results to the growth control.

Generally, the HELST obtained better results in all the performed tests, which is interesting from the point of view of the conservation of the species, as since the leaves are more accessible, the collection is practical and less harmful to the plant.

## 3. Discussion

Phytochemical studies using the *S. tuberosa* leaf methanolic and ethyl acetate extracts also revealed the presence of phenols, flavonoids, flavones, triterpenes, and steroid compounds, similar to those found in the present study analysis; however, these also found cinnamic derivatives, saponins, and leucoanthocyanidins, which differ from the present results [14,24]. The *S. monbin* ethanolic extract revealed the presence of polyphenols and flavonoids [25]. The authors of [9] found chlorogenic acid in *S. tuberosa* leaf hydroethanolic extract by HPLC analysis, with the same compound being identified in the root hydroalcoholic extract of the same species.

The authors of [26] inhibited *C. albicans* growth with *S. tuberosa* leaf hexanic extract using a different methodology, obtaining an IC_50_ of 2.0 mg/mL, a result that is different to those of the present study; however, the authors did not detect a fungicidal effect, a result similar to that of this study. *S. tuberosa* bark ethanolic extract did not inhibit growth of the *C. albicans*, *C. glabrata*, *C. krusei*, or *C. tropicalis* strains with the disc diffusion technique [27], whose effect may be explained by the fact that the hexane extract have a lower polarity and the extracted constituents are different from those obtained with the extracts in this study [28].

Some of the compounds identified in the *S. tuberosa* leaf and root extracts are reported in the literature for their antifungal activity. The author of [29] reports the antifungal activity of mangiferin. An inhibition in filament growth was observed with the *C. albicans* (ATCC 90028 and MTCC 186) strains and clinical isolates (CA1, CA2, CA3, and CA4) in a study addressing the synergistic association of quinic acid with undecanoic acid on *Candida* spp. morphological transition. However, only a moderate inhibition was observed for *C. tropicalis* (MTCC 184) strains and isolates (CT1, CT2, and CT3) [30]. The authors of [31] tested several compounds using the disc diffusion method where one of them was narigenin, a compound present in HERST, which demonstrated activity against *C. albicans*.

The authors of [30] showed that quinic acid, which was identified in the HELST, did not inhibit the growth of *Candida* strains at varying concentrations (12.5–800 µg mL^−1^). In a study on the antifungal activity of chlorogenic acid, the yeasts *Trichosporon beigelii* (KCTC 7707), *Malassezia furfur* (KCTC 7744), and *C. albicans* (TIMM 1768) were susceptible to the compound, with MIC values between 40 and 80 µg/mL [32]. Chlorogenic acid achieved a partial reduction in the mycelial growth of some pathogenic plant fungi [33]. The chlorogenic acid effect on *C. albicans* dimorphism was investigated, where this was able to inhibit and destroy hyphae. The authors attribute the action of this compound, which affects yeast cells, to it damaging their membranes and disrupting their membrane potential [32].

The use of different *Spondias monbin* L. parts in combination with fluconazole inhibited the growth of *C. albicans* ATCC 90029 and *C. krusei*, where the author associated this result with the compounds present in the plant, which may have increased the effect of the drug [34]. The *S. monbin* leaf extract inhibited the *Saccharomyces cerevisiae* NCPF 3178 yeast strain (7 to 7.5 mm) in the disk diffusion method and presented a minimum inhibitory concentration (MIC) of 250 µg/mL [35] by microdilution. The authors of [25] tested *S. monbin* ethanolic extract against *C. albicans* ATCC 10231, *C. guilliermondii* ATCC 6260 and *C. krusei* ATCC 34135 using a similar technique to that applied in this study, obtaining MIC values >1000 µg/mL^−1^.

Other species that are also from the Anacardiaceae family have been investigated for their activity against fungi. A study with *Anacardium occidentale* L. and *Mangifera indica* L. leaves against *C. albicans* obtained an MIC of 1250 µg/mL and MFC of 1250 and 2500 µg/mL, respectively [36]. The *A. occidentale* ethanolic extract from the plant’s flowers, leaves, and stem bark were investigated using the disc diffusion technique, where the flower extract inhibited different *C. albicans* and *C. tropicalis* (1 mm) strains, while the leaf and stem bark extracts inhibited *C. albicans* 37 (1 mm) and fluconazole inhibited the *C. albicans* and *C. tropicalis* strains (1 to 2 mm) [37].

The diversity of chemical classes that are present in an extract are defined as complex heterogeneous mixtures, each of which has different biological or pharmacological activities and collectively contribute to the bioactivity of the extract as a whole [38,39], thus the moderate activity demonstrated by the *S. tuberosa* leaf and root hydroalcoholic extracts against species from the *Candida* genus may have occurred due to the synergistic action of the chemical constituents present in the extracts.

## 4. Materials and Methods

### 4.1. Botanical Material Collection and Identification

The botanical material was collected with consent from the Biodiversity Authorization and Information System SISBIO-number 64293-1, in the community of Lameiro (07°, 15′03.1′′ S and 39°, 23′48.3′′ W of Greenwich), in the municipality of Crato, southern Ceará, Brazil. Specimens from seven individuals were collected from 8:00 to 9:30 in the morning. A sample of the collected material was deposited in the Herbarium Dárdano de Andrade Lima (HCDAL) of the Regional University of Cariri-URCA with the herbarium number 13.728, identified by Professor Ana Cleide Alcantara Morais Mendonça as being the species *Spondias tuberosa* Arruda.

### 4.2. Extract Acquisition

For the extract preparations, healthy young roots and leaves collected from the upper part of the plant were used. The hydroalcoholic extract (tincture or alcohol) was prepared in 70% alcohol (ethanol) at 500 g of fresh leaves or 400 g of dried root for every 2.652 L of alcohol/water proportion [40] with modifications.

Extract drying was performed by spray drying using the Mini-spray dryer MSDi 1.0 (Labmaq do Brasil) using a 1.2-mm spray nozzle under the following operating conditions: (a) Flow control: 500 mL/h; (b) inlet temperature: 130 ± 2 °C; (c) outlet temperature: 74 ± 2 °C; (d) atomization air flow: 45 L/min; and (e) blower flow: 1.95 m^3^/min. The spray drying process consists of a product that is in the liquid state changing to a solid state in powder form by passing through a heated medium under continuous operation [41].

### 4.3. Chemical Analysis

#### 4.3.1. Preliminary Phytochemical Analysis

The extracts were subjected to preliminary phytochemical analysis based on qualitative methods proposed by [42]. In these experiments, characterization of the main metabolite classes was performed by chemical reactions, with the addition of specific reagents resulting in color changes and/or precipitate formations characteristic of the following metabolite classes: Alkaloids, steroids, phenols, flavonoids, tannins, and triterpenoids. All experiments were performed in triplicates and the secondary metabolite classes present in the extracts were classified as present (+) or absent (-).

#### 4.3.2. Compound Identification by Ultra-Performance Liquid Chromatography Coupled to Quadrupole/Time of Flight System (UPLC-MS-ESI-QTOF)

Identification of the compounds present in the extracts was performed in an Acquity^®^ UPLC system coupled to a Quadrupole/Time of Flight (QTOF) system (Waters Corporation, Milford, MA, USA), provided by the Chemical and Natural Products Laboratory, Embrapa Tropical Agroindustry (Fortaleza, Ceará). Chromatographic runs were performed with a Waters Acquity^®^ UPLC BEH column (150 × 2.1 mm; 1.7 µm), a fixed temperature of 40 °C, mobile phases with 0.1% formic acid (A) and acetonitrile with 0.1% formic acid (B), gradient ranging from 2% to 95% B (15 min), 0.4 mL/min flow rate, and 5 µL injection volume. The ESI^-^ mode was obtained in the 110–1180 Da range with a fixed source temperature of 120 °C, 350 °C desolvation temperature, 500 L/h desolvation gas flow, 0.5 V extraction cone, and 2.6 kV capillary voltage. Leucine encephalin was used as the lock mass. The MSE (high energy mass spectrometry) acquisition mode was used. The instrument was controlled by the Masslynx^®^ 4.1 software (Waters Corporation, Milford, USA).

### 4.4. Antifungal Assays

#### 4.4.1. Microorganisms, Culture Media, Inoculum Preparation, and Drugs and Reagents Used

Two standard strains, *Candida albicans*-CA INCQS 40006 and *Candida tropicalis*-CT INCQS 40042, obtained from the Oswaldo Cruz Culture Collection of the National Institute for Quality Control in Health (INCQS) and two clinical isolates, *Candida albicans*-CA URM 5974 and *Candida tropicalis*-CT URM 4262, provided by the Recife Micoteca University (URM) from the Federal University of Pernambuco–UFPE were used to evaluate antifungal activity.

The Sabouraud Dextrose Agar (SDA) solid medium, prepared according to the manufacturer’s instructions, and the doubly concentrated Sabouraud Dextrose Broth (SDB) liquid medium, purchased from HIMEDIA^®^, were used. The Potato Dextrose Agar (PDA) medium, purchased from Difco^®^, depleted by dilution and added to agar was used for the fungal morphology analysis. The media were solubilized with distilled water and autoclaved at 121 °C for 15 min.

All strains were initially kept in refrigerated (8 °C) slanted SDA at the Cariri Applied Mycology Laboratory-LMAC of the Regional University of Cariri-URCA. For microdilution assays, strains were initially cultivated on SDA media at 37 °C for 24 h. Cell suspensions were then prepared in tubes containing 4 mL of sterile saline solution (0.9% NaCl) and their turbidity was compared and adjusted according to the MacFarland 0.5 scale [43].

Dimethyl sulfoxide (DMSO-Vetec) was used for the dilution of the extracts and the antifungal fluconazole 150 mg (prati-donaduzzi), diluted in water, was used as the commercial drug reference in the tests. For the initial extract solution preparations, 0.15 g were weighed and solubilized in 1 mL of DMSO. The extracts and fluconazole were then further diluted in sterile distilled water to obtain the desired test concentration (16,384 µg/mL).

#### 4.4.2. IC_50_ Determination and Cell Viability Curve

This assay was performed using the broth microdilution technique in 96-well plates. Each well was filled with the medium and inoculum, followed by the extracts and fluconazole, before being individually microdiluted (81,92 to 8 µg/mL). The last well was reserved as a growth control, all tests were performed in quadruplicate [44], with changes in concentration. Natural product and fluconazole dilution controls (with 0.9% sodium chloride solution instead of inoculum) and sterility controls were also performed. The plates were incubated at 37 °C for 24 h, then read on an ELISA spectrophotometer (Kasuaki-DR-200Bs-BI) at a wavelength of 450 nm. Results from the absorbance were used to construct the cell viability curve; from the mean cell viability curve, the IC_50_ values of the extracts and fluconazole were calculated [45].

#### 4.4.3. Minimum Fungicidal Concentration (MFC) Determination

For this test, a sterile rod was introduced into each well of the microdilution test plate (except for the sterility control), where after homogenization of the medium contained in the well, a petri dish with SDA was sub-cultured by transferring a small aliquot of the test solution (medium + inoculum + natural product). The plates were incubated at 37 °C for 24 h and checked for *Candida* colony growth or lack thereof [46], with modifications. The MFC was defined as the lowest natural product concentration capable of inhibiting the growth of fungal colonies.

#### 4.4.4. Evaluation of the Modifying Effect of Fluconazole Action

The combination of the extracts with the reference drug (fluconazole) was performed to verify whether the antifungal action was modified by the presence of the extract in the medium. Solutions containing the extracts were tested at a sub-inhibitory concentration (MFC/16), according to the methodology used by [47], with changes in concentrations. If the extracts potentiated the action of the antifungal, the observed effect was considered to be synergistic; however, if it impaired its action, it was considered to be an antagonistic effect. Fluconazole was microdiluted at a 1:1 proportion up to the penultimate well. Sterility controls were also performed. All tests were performed in quadruplicate. The plates were incubated at 37 °C for 24 h. The readings were performed in an ELISA spectrophotometry device (Kasuaki-DR-200Bs-BI) [45].

#### 4.4.5. Effect of the Extracts and Fluconazole on *Candida* Morphological Transition

Microculture chambers were used to verify changes in fungal morphology by the extracts and fluconazole through the reduction or prevention of hyphae development. The extracts and fluconazole were individually evaluated at different concentrations (HCE-8192 µg/mL, HCE/4-2048 µg/mL, and HCE/16-512 µg/mL, where HCE is the highest concentration evaluated by microdilution). The tests were performed according to [48,49], with modifications: Two parallel striae were made in the solid medium (PDA), which were subsequently covered with a sterile cover slip. For comparative purposes, growth controls as well as fluconazole antifungal controls were performed. The chambers were incubated for 24 h (37 °C), inspected, and recorded by an optical microscope (AXIO IMAGER M2-3525001980-ZEISS-Germany) using a 20X objective. The Zen 2.0 software was used to measure hyphae and pseudohyphae extensions, where images were taken from each complete stria, with five random images being selected from these, according to the concentration. The values were used for statistical analysis [50].

### 4.5. Statistical Analysis

A two-way ANOVA was applied to each sample, comparing the values for each extract concentration, with Bonferroni’s post hoc test, in which *p* < 0.05 and *p* < 0.0001 were considered significant while *p* > 0.05 was not significant. IC_50_ values were obtained by nonlinear regression with unknown interpolation of standard curves obtained from fungal growth values as a function of the extract concentration, expressed in μg/mL. Statistical analysis was performed using the Graphpad Prism software, version 6.0.

Measurement of the complete striae borders, as well as of the regions where hyphal growth was observed, was performed for virulence analysis. Then, measurements of all hyphal filaments identified in five randomly selected regions from each striae and concentration were performed. Hyphal filament length was averaged and analyzed by ANOVA followed by Bonferroni’s correction for multiple comparisons according to the product concentration [50].

## 5. Conclusions

Preliminary phytochemical analysis of the extracts detected the presence of phenolic and flavonoid compounds in both extracts. The activity of the extracts against the tested strains was not clinically relevant since direct contact growth inhibition only began at high concentrations. In the fluconazole modulatory assay, the extracts presented synergistic effects with some concentrations. The natural product inhibited hyphal and pseudohyphal growth in the fungal morphological transition assay, depending on the tested concentrations.

Studies on the modulatory and morphological activity of *S. tuberosa* extracts are rare, with the present study being the first to report on these activities, as well as to report on the analysis of *S. tuberosa* extracts by high performance liquid chromatography. Thus, research addressing the mechanisms of action of these extracts and the bioactivity of their chemical constituents is necessary to better explore the pharmacological potential of the species.

## Figures and Tables

**Table 1:** Preliminary phytochemical analysis of extracts.

	Special Metabolite Classes (SMC)									
	SMC 1	SMC 2	SMC 3	SMC 4	SCM 5	SMC 6	SMC 7	SMC 8	SMC 9	SMC 10
HELST	+	-	+	-	-	+	-	+	-	+
HERST	+	-	-	+	-	+	-	-	+	-

SMC 1: Phenols; SMC 2: Tannins; SMC 3: Flavonoids of the flavone, flavonol and xanthone type; SMC 4: Flavonoids of the anthocyanin and anthocyanidin type; SMC 5: Flavanoid of leucoanthocyanidins, catechins; SMC 6: Flavanone-type flavanoid; SMC 7: Flavanoid Flavanoid; SMC 8: Steroids; SMC 9: Triterpenoids; SMC 10: Alkaloids; (+): positive; (-): absent.

**Table 2:** Ultra-Performance Liquid Chromatography Coupled to Quadrupole/Time of Flight System (UPLC-MS-ESI-QTOF) identification of hydroalcoholic extract compounds of *Spondias tuberora* leaves.

Peak no.	Rt min	[M-H]^-^ Observed	[M-H]^-^ Calculated	Product Ions (MS/MS)	Empirical Formula	Ppm (error)	Putative Name	References
**1**	2.82	174.9525	174.9502	175.9599	C_6_H_6_O_6_		Dehydroascorbic acid	[15]
**2**	3.28	443.1294	443.1283	381.1805, 281.1358, 119.0361	C_21_H_32_O_10_	2.5	Dehydrophaseic acid hexose	[16]
**3**	3.40	189.0021	189.0035	207,0115, 188.9965, 126.9987	C_6_H_5_O_7_	−7.4	None identified	-
**4**	3.65	188.9986	188.9977	207.0199, 188.9996, 127.0019	C_13_HO_2_	4.8	None identified	-
**5**	4.53	191.0109	191.0133	173.0243, 127.0278, 85.0283	C_7_H_12_O_6_	−12.6	Quinic acid (Organic acid)	[17,18]
**6**	4.65	343.0833	343.0818	191.0497, 169.0125, 125.0265	C_14_H_16_O_10_	4.4	Galloyl quinic acid isomer Ia	[18]
**7**	4.67	343.0508	343.0513	191.0523, 169.0112, 125.0259	C_14_H_16_O_10_	−1.5	Galloyl quinic acid isomer IIa	[18]
**8**	4.74	343.1045	343.1029	191.0196, 169.0011, 125.9942	C_14_H_16_O_10_	4.9	Galloyl quinic acid isomer III	[18])
**9**	4.89	343.0904	343.0877	191.0540, 169.0121, 125.0247	C_14_H_16_O_10_	7.9	Galloyl quinic acid isomer IV	[18]
**10**	6.28	421.2582	421.2590	331.0801, 3010357	C_19_H_18_O_11_	−1.9	Mangiferin	[15,19]
**11**	6.48	939.4997	939.4989	787.4410, 277.2166, 125.0243	C_41_H_32_O_26_	0.9	Penta-O-galloyl hexoside	[20]
**12**	6.56	397.1313	397.1287	502.2910, 474.2616, 277.2155	C_22_H_21_O_7_	6.5	None identified	-
**13**	6.90	277.2142	277.2168	279.2315, 277.2151, 189.0038	C_18_H_29_O_2_	−9.4	None identified	-
**14**	7.00	339.2007	339.2019	-	C_22_H_28_O_3_	−3.5	Caffeoyl-D-glucose	[16]
**15**	7.50	483.2540	483.2535	271.0134, 169.0511, 125.9984	C_20_H_20_O_14_	1.0	Digalloyl glucose (Digalloyglucose)	[16,21]

[M-H]^-^—Reference Ion on negative ion mode.

**Table 3:** UPLC-MS-ESI-QTOF identification of hydroalcoholic extract compounds of *Spondias tuberora* roots.

Peak no.	Rt min	[M-H]^-^ Observed	[M-H]^-^ Calculated	Product Ions (MS/MS)	Empirical Formula	Ppm (error)	Putative Name	References
**1**	2.83	272.9554	272.9578	274.9536, 273.9563, 158.9753	C_15_H_12_O_5_	−8.8	(±)-Naringenin	[15]
**2**	3.11	341.2104	341.2117	297.2246, 295.2048, 119.0465	C_22_H_30_O_3_	2.1	Anacardic acid 1	[16]
**3**	3.14	343.1186	343.1182	299.2344	C_22_H_21_O_3_	1.2	Anacardic acid 2	[16]
**4**	3.16	377.0822	377.0814	379.0782, 377.0770, 341.1009	C_25_H_13_O_4_	2.1	No identified	-
**5**	3.22	345.0010	345.0035	301.2471	C_22_H_34_O_3_	−7.2	Anacardic acid 3	[16]
**6**	3.52	355.0232	355.0243	355.0258, 163.0371	C_16_H_18_O_9_	−3.1	Chlorogenic acid	[9]
**7**	4.39	411.0173	411.0213	411.0232, 240.9987, 169.0123	C_17_H_7_N_4_O_9_	−9.7	None identified	-
**8**	4.50	461.1219	461.1236	257.0848, 229.8624, 151.0033	C_22_H_21_O_11_	−3.7	Kaempferol-7-Oglucuronide	[16]
**9**	4.62	197.0424	197.0450	199.06, 198.05, 182.0187	C_9_H_10_O_5_	−13.2	2-Hydroxy-3,4-dimethoxybenzoic acid	[15]
**10**	4.90	433.0583	433.0560	300.9944, 271.0716	C_20_H_18_O_11_	5.3	Quercetin O-pentoside	[20]
**11**	4.99	315.0096	315.0082	394.9633, 315.0095, 299.9882	C_16_H_12_O_7_	4.4	Rhamnetin	[22]
**12**	5.05	463.1048	463.1029	316.0255, 271.0625	C_21_H_20_O_12_	4.1	Myricetin O-deoxyhexoside	[20]
**13**	5.08	449.1247	449.1236	316.0205	C_20_H_18_O_12_	2.4	Myricetin O-pentoside	[20]
**14**	5.35	331.2458	331.2484	271.0428, 241.0313, 125.0252	C_9_H_16_O_13_	−7.8	Monogalloyl-glucose	[16]
**15**	5.52	461.2616	461.2598	315.0131	C_21_H_17_O_12_	3.9	Isorhamnetin Orhamnoside	[23]
**16**	6.15	833.5256	833.5262	833.5175, 507.2970, 175.0390	C_43_H_77_O_15_	0.7	None identified	-

[M-H]^-^—Reference Ion on negative ion mode.

**Table 4:** 50% inhibitory concentration (IC_50_) of microorganisms (µg/mL) by extracts, fluconazole, and their combination.

	CA INCQS 40006	CA URM 5974	CT INCQS 40042	CT URM 4262
HELST	6211.1 *	5716.3 *	7166.8 *	7805.8 *
HERST	6264.8 *	8919.9 *	51070.9 *	6175.4 *
FLUCONAZOLE	22.79	3.97	88.08	47.30
HELST + FCZ	13.60	2.65 *	44.86 *	43.75
HERST + FCZ	25.81	4.38	278.41 *	26.15 *

CA: *Candida albicans*; CT: *Candida tropicalis*; INCQS: National Institute of Quality Control in Health; URM: University Recife Mycology; HELST: Hydroalcoolic Extract of Leaves *Spondias tuberosa*; HERST: Hydroalcoholic Extract of the Roots of *Spondias tuberosa*; FCZ: Fluconazole. * Corresponding to the ANOVA with *p* < 0.0001 for all data.

**Table 5:** Minimal fungicidal concentration (MFC) of microorganisms (µg/mL) by extracts, fluconazole, and their combination.

	CA INCQS 40006	CA URM 5974	CT INCQS 40042	CT URM 4262
HELST	≥16,384	≥16,384	≥16,384	≥16,384
HERST	≥16,384	≥16,384	≥16,384	≥16,384
FLUCONAZOLE	8192	8192	≥16,384	≥16,384
HELST + FCZ	8192	2048	≥16,384	8192
HERST + FCZ	8192	4096	≥16,384	≥16,384

CA: *Candida albicans*; CT: *Candida tropicalis*; INCQS: National Institute of Quality Control in Health; URM: University Recife Mycology; HELST: Hydroalcoolic Extract of Leaves *Spondias tuberosa*; HERST: Hydroalcoholic Extract of the Roots of *Spondias tuberosa*; FCZ: Fluconazole.
